# Reliability of the International Spinal Cord Injury Physical Therapy–Occupational Therapy Basic Data Set

**DOI:** 10.1089/neur.2024.0020

**Published:** 2024-07-01

**Authors:** Edelle C. Field-Fote, Kim D. Anderson, Maclain Capron, Ruediger Rupp, Linda Jones, Mary Schmidt-Read, Vanessa K. Noonan, Anne Bryden, Sara Mulroy, Walter Weiss, Mario Widmer, Henrik Hagen Poder, Vivien Jørgensen, Eimear Smith, Mariel Purcell, Fin Biering-Sørensen

**Affiliations:** ^1^ Crawford Research Institute, Shepherd Center, Atlanta, Georgia, USA.; ^2^ Division of Physical Therapy, Emory University School of Medicine, Atlanta, Georgia, USA.; ^3^ Program in Applied Physiology, School of Biological Sciences, Georgia Institute of Technology, Atlanta, Georgia, USA.; ^4^ MetroHealth Rehabilitation Institute, MetroHealth System, Cleveland, Ohio, USA.; ^5^ Department of Physical Medicine and Rehabilitation, Case Western Reserve University, Cleveland, Ohio, USA.; ^6^ Department of Rehabilitation Medicine, Thomas Jefferson University, Philadelphia, Pennsylvania, USA.; ^7^ Spinal Cord Injury Center, Heidelberg University Hospital, Heidelberg, Germany.; ^8^ Thomas Jefferson University, Philadelphia, Pennsylvania, USA.; ^9^ Magee Rehabilitation Hospital, Jefferson Health, Philadelphia, Pennsylvania, USA.; ^10^ Praxis Spinal Cord Institute, Vancouver, British Columbia, Canada.; ^11^ International Collaboration on Repair Discoveries, Vancouver, British Columbia, Canada.; ^12^ Institute for Functional Restoration, Case Western Reserve University, Cleveland, Ohio, USA.; ^13^ Pathokinesiology Laboratory, Rancho Los Amigos National Rehabilitation Center, Downey, California, USA.; ^14^ Department of Therapy, Swiss Paraplegic Centre, Nottwil, Switzerland.; ^15^ Neuro-Musculoskeletal Functioning and Mobility, Swiss Paraplegic Research, Nottwil, Switzerland.; ^16^ Department for Brain and Spinal Cord Injuries, Bodil Eskesen Centre, Neuroscience Centre, Rigshospitalet, University of Copenhagen, Copenhagen, Denmark.; ^17^ Sunnaas Rehabilitation Hospital, Bjørnemyr, Norway.; ^18^ National Rehabilitation Hospital, Dublin, Ireland.; ^19^ National Spinal Injuries Unit, Queen Elizabeth University Hospital, Glasgow, United Kingdom.; ^20^ Department of Clinical Medicine, University of Copenhagen, Copenhagen, Denmark.

**Keywords:** neuroplasticity, outcome measures, recovery, rehabilitation, spinal cord injury

## Abstract

In interventional clinical trials for persons with spinal cord injury (SCI), the influence of experimental biological, pharmacological, or device-related interventions must be differentiated from that of physical and occupational therapy interventions, as rehabilitation influences motor-related outcomes. The International Spinal Cord Injury (ISCI) Physical Therapy**–**Occupational Therapy Basic Data Set (PT-OT BDS) was developed with the intent to track the content and time of rehabilitation interventions that are delivered concurrently with experimental interventions. We assessed the reliability of the PT-OT BDS based on agreement between users. Following an online training session, physical therapists (PTs) and occupational therapists (OTs) from 10 SCI clinical centers across 7 countries participated. At each center, pairs of therapists (a treating therapist and an observing therapist; PT/PT, OT/OT, or PT/OT) used the PT-OT BDS to record the content and time of therapy sessions for 20 patients. Data were analyzed to determine agreement between therapist pairs regarding the content of the therapy session. The influence of therapist characteristics (professional discipline [PT/OT], years of experience working with individuals with SCI), patient characteristics (level [tetraplegia/paraplegia] and severity [complete/incomplete] of injury), setting (inpatient/outpatient), and whether the center was U.S.- versus non-U.S.-based were also analyzed. There was high agreement for five of seven categories and medium agreement for the remaining two categories. For six of the seven intervention categories, there were no significant differences between the treating and the observing therapists in the percentage of instances that a specific category was selected. Characteristics of the therapists, characteristics of the patient, therapy setting, and global location of the center had no meaningful influence on level of agreement between therapist pairs. The BDS is reliable for use across settings, countries, and with patients of various impairment levels. The study also helped identify additional areas where refinement of the syllabus would be of value.

## Introduction

Pre-clinical evidence in animal models of spinal cord injury (SCI) indicates that physical activity, practice, and training influence the motor outcomes of randomized intervention studies.^1–4^ In humans with SCI, direct assessment of the efficacy of standard rehabilitation via randomized comparison of outcomes among patients with SCI who do and do not receive rehabilitation is precluded by ethical considerations. However, a systematic review of experimental interventions for persons with SCI concluded that clinical trials that include a physical therapy (PT) and/or occupational therapy (OT) component are more likely to have meaningful effects on motor-related outcomes.^5^

As rehabilitation influences motor-related outcomes, in interventional clinical trials, the influence of the experimental biological, pharmacological, or device-related intervention must be differentiated from that of physical and occupational therapy interventions. The International Spinal Cord Injury Physical Therapy–Occupational Therapy Basic Data Set (ISCI PT-OT BDS, v.1.2) was developed with the intent to track the content and time of rehabilitation interventions that are delivered concurrently with experimental interventions.^6^ The ISCI PT-OT BDS enables standardized documentation of PT and OT interventions delivered in the clinical setting as part of a controlled clinical trial that is intended to improve voluntary motor function. PT and OT interventions are assigned to seven categories, of which five are activity-directed (bed/seated activities; standing activities; walking, stairs [inside, outside]; gross motor upper extremity [UE]; fine motor UE), and two are impairment-directed (strength training and/or electrical stimulation administered to increase strength; endurance training and/or electrical stimulation administered to increase endurance).

When the ISCI PT-OT BDS is used to document therapy interventions that are part of a clinical trial, the individual documenting the content of a therapy intervention may be a PT and/or an OT. Differences in training and clinical emphases between the disciplines of PT and OT may result in different perceptions of the intervention being delivered to a patient. Likewise, differences in perceptions about an intervention may be attributable to therapist years of experience working with individuals with SCI.

Apart from therapist-related factors, there are factors related to the patient whose treatment session is being documented that may account for differences in therapist perception of the intervention. The content of PT and OT sessions is likely to be largely determined by the degree of impairment of the individual with SCI. Different types of intervention are appropriate based on whether the individual being treated has motor-complete tetraplegia, motor-incomplete tetraplegia, motor-complete paraplegia, or motor-incomplete paraplegia. As such, the way the content and time of the therapy session are documented may be influenced by the degree of impairment of the individual whose therapy session is being documented.

Differences in the setting in which a patient with SCI is being treated may result in differences in what is documented in inpatient versus outpatient settings. Differences between U.S.-based versus non-U.S.-based therapists in what content is emphasized during therapy may be a source of differences in documentation of therapy content. Finally, diferences in the types of interventions that are done, in the equipment, and in the amount of time allocated to treatment must also be considered.

The purpose of this study was to determine whether the ISCI PT-OT BDS could be used to reliably capture information about the content of a therapy session. To address this question, we assessed the inter-rater reliability of information collected by pairs of therapists who had been trained to use the ISCI PT-OT BDS based on instructions in the syllabus. We assessed the level of agreement between therapist pairs in terms of therapy category selection and time spent in each category. The documentation was intended to reflect interventions delivered to a patient with SCI during a regularly scheduled treatment session.

## Methods

To the extent possible, the methods adhered to the recommendations of the ISCI Data Set Committee.^7^ The ISCI PT-OT BDS work group developed the study syllabus (ISCI PT-OT Basic Data Set syllabus v1.2)^6^ and recruited 10 SCI centers in 7 countries (4 in the United States and 1 each in Denmark, Germany, Ireland, Norway, Switzerland, and the United Kingdom) for participation in the study. All centers met the ethics requirements of their respective country/institution before the initiation of study activities at their respective center. Each participating center verified that their center offered the full range of interventions described in the BDS. Each center had one site leader serving as the liaison to the work group. The site leaders recruited PTs and OTs who had experience in the rehabilitation of persons with SCI at their respective center to participate in the study. Several online sessions were held to review the study syllabus with the site leaders and therapists and to clarify any areas of uncertainty about the syllabus.

At each center, data were recorded during the course of regularly scheduled therapy sessions with 20 different patients with SCI. All patients were at least 18 years of age, had injury etiology of either traumatic or nontraumatic SCI of any postinjury time point, and were receiving therapy in either the inpatient or the outpatient setting. Each participating center endeavored to provide data that reflected the spectrum of SCI level and severity, including from individuals with motor-complete tetraplegia (American Spinal Injury Association Impairment Scale [AIS] A & B), motor-incomplete tetraplegia (AIS C & D), motor-compete paraplegia (AIS A & B), or motor-incomplete paraplegia.^8^ To ensure that the data collected reflected the broadest possible range of characteristics of individuals with SCI, each individual with SCI could be observed only once. To be included in the data analysis, the observed intervention session had to be at least 30 min in duration.

Therapist raters were either PTs or OTs, with any level of experience in SCI rehabilitation. Each participating site had at least three therapists who contributed data, and centers were encouraged to have both PTs and OTs participate in the study. For each therapy session in which data were collected, therapists worked in pairs comprising a treating therapist and an observing therapist; pairs could comprise PT/OT, PT/PT, or OT/OT. It was deemed acceptable to have a single therapist be the observer for all observations, so long as there are at least two other treating therapists.

Data were collected using the ISCI PT-OT BDS v.1.2, the treating therapists recorded the intervention category and amount of time they believed was spent in the activity- or impairment-directed intervention when they transitioned from one intervention to another. If an intervention qualified to be considered in both an activity-directed and an impairment-directed category, then therapists were instructed to score it only in the activity-directed category. Therapists reported time spent on a specific intervention by 15-min intervals (<15 min, 15–29 min, 30–44 min, 45–59 min, or ≥ 60 min). The observing therapist observed the entire session, using a timer to time the interventions, and recorded the time spent in the categories he/she considered appropriate. The treating therapist and observing therapist were blinded to each other’s entries.

Participating centers were provided with a formatted electronic data workbook (Excel) in which to enter their center’s data for analysis. Each site also sent electronic (scanned) versions of the paper data collection forms, and members of the work group performed random fidelity checks for quality control on 10% of the records based on a random assignment.

In addition to information about the therapy session, the participating therapists indicated on the data collection form information about the following: (1) whether they were the treating therapist or observer; (2) their professional discipline (PT, OT); (3) their years of experience in working with individuals with SCI (<3 years, ≥3 years); (4) degree of impairment of the individual with SCI who is receiving the intervention (motor-incomplete tetraplegia, motor-complete tetraplegia, motor-incomplete paraplegia, motor-incomplete paraplegia); (5) therapy setting (inpatient, outpatient); and (6) and unique center code (to identify center as U.S.-based vs. non-U.S.-based in analysis).

### Data analyses

Agreement between observing and treating therapists was assessed on a categorical level and a time level. If both therapists provided any amount of time for a category, then they were deemed to have agreed that the category occurred during the session. Agreement regarding time was characterized as providing either the same time interval or one time interval difference for a specific intervention. For example, instances in which the treating therapist selected 15–29 min for an intervention while the observing therapist selected 30–44 min, the therapists are considered to be in agreement. Disagreement was determined by any instances in which therapists indicated a difference of two or more time intervals for any given intervention. If a therapist did not report a time interval for a specific intervention during the therapy session being assessed, and the other therapist did report a time interval for a specific intervention during that therapy session, then this was deemed to be a disagreement. Instances in which neither therapist recorded a specific intervention were not included in the agreement analyses to avoid overinflation of agreement percentage.

Percent agreement and intraclass correlation coefficients (ICCs) were calculated to assess agreement among therapists across interventions for intervention time intervals. Percent agreement of 75% or greater was considered high, 50–75% was considered medium, and less than 50% was considered low. ICC values were interpreted as follows: ICC ≥ 0.90 indicating excellent agreement, 0.75 ≤ ICC < 0.90 indicating good agreement, 0.50 ≤ ICC < 0.75 indicating moderate agreement, and ICC < 0.5 indicating poor agreement.^9^ One-way random-effects models were used to account for the study consisting of multiple sets of raters across sites.

Differences between treating and observing therapists in terms of time intervals for interventions were investigated using independent sample *t*-tests. For the purpose of this analysis, time intervals were treated as continuous data with 1 = <15 min, 2 = 15–29 min, and so on. Response proportion, the proportion of instances in which the treating or observing therapist reported an intervention, was also assessed. Instances where a therapist reported any amount of time were coded as reporting that the intervention occurred, regardless of duration.

Percent agreement and Pearson chi-square analyses were conducted to examine differences between therapist pairs because of discipline (PT, OT), years of experience (<3 years, ≥3 years), degree of impairment (motor-complete tetraplegia, motor-incomplete tetraplegia, motor-complete paraplegia, motor-incomplete paraplegia), therapy setting (inpatient, outpatient), and global location of the center (U.S.-based, non-U.S.-based).

## Results

At each of the 10 centers, 20 therapy sessions were observed by pairs of therapists resulting in a total of 400 observations. Fidelity checks revealed errors in the data of two centers, prompting a full check of data from all centers. Any errors identified in the transfer of data from the data collection forms to the electronic forms were corrected.

### Therapist and patient characteristics

Of the 400 observations, 68.5% were conducted by PTs, whereas 31.5% were conducted by OTs. The PT-OT providers averaged 7.7 years of experience (standard deviation [SD] = 8.46). Among the patients being observed, 41% had motor-incomplete tetraplegia, 13% had motor-complete tetraplegia, 26.5% had motor-incomplete paraplegia, and 19.5% had motor complete paraplegia.

### Agreement on intervention category and time

As displayed in Table 1, the category agreement between the treating and observing therapist pairs was high (75% or greater) for five of the seven intervention categories. The exceptions were gross UE and endurance training, in which agreement between the treating and the observing pairs was medium (≥50% and <75%). Time agreement between the treating and observing therapist pairs was high for three of the seven intervention categories, including bed/seated control activities, walking/stairs, and strength training. The exceptions were standing control, gross UE, fine motor UE, and endurance training, in which percent agreement between the treating and the observing pairs was medium. ICC estimates demonstrated moderate-to-good reliability for all intervention categories with the exceptions of gross UE training and endurance training.

**Table 1.** Intervention (A–E Activity Based; F–G Impairment Based) Agreement

Intervention	Observations included^a^	Category agreement percentage	Time agreement percentage	ICC (95% CI)^b^
A Bed/seated control activities	125	80.0	79.2	0.815 (0.736, 0.870)
B Standing control activities	74	75.7	74.3	0.549 (0.285, 0.716)
C Walking, stairs (inside, outside)	48	93.8	93.8	0.748 (0.553, 0.859)
D Gross motor upper extremity	59	66.1	65.5	0.470 (0.112, 0.685)
E Fine motor upper extremity	41	78.0	73.2	0.728 (0.492, 0.854)
F Strength training (including electrical stimulation for strength)	109	78.9	78.0	0.656 (0.497, 0.764)
G Endurance training (including electrical stimulation for endurance)	53	50.9	50.9	0.105 (−0.546, 0.482)

^a^ Number of data entries for which at least one rater provided data for duration of intervention.

^b^ Run as a one-way random-effects model.

CI, confidence interval; ICC, intraclass correlation coefficient.

### Treating and observing therapist interactions

As shown in Table 2, for six of the seven intervention categories, there were no significant differences between the treating and the observing therapists in the percentage of instances that a specific category was selected. However, the treating and observing therapists did differ in the percentage of instances where endurance training category was selected (25% vs. 15%, for the treating and observing therapists, respectively). Relative to the treating therapist, the observing therapists selected the category of endurance training significantly less often. Reported time spent on a category did not differ between the treating and the observing therapists for any of the seven categories.

**Table 2.** Treating and Observing Therapist Interactions

Intervention	Therapist role	Response proportion	Chi-square	Time interval selection (mean, SD)	*t*-Test
A Bed/seated control activities	Treating therapist	52.5	*p* = 0.131	1.9, 0.87	*p* = 0.244
	Observing therapist	60.0		1.76, 0.89	
B Standing control activities	Treating therapist	33.0	*p* = 0.831	1.38, 0.60	*p* = 0.262
	Observing therapist	32.0		1.27, 0.54	
C Walking, stairs (inside, outside)	Treating therapist	23.0	*p* = 0.906	1.52, 0.69	*p* = 0.691
	Observing therapist	23.5		1.57, 0.58	
D Gross motor upper extremity	Treating therapist	22.0	*p* = 0.245	1.52, 1.00	*p* = 0.666
	Observing therapist	27.0		1.44, 0.79	
E Fine motor upper extremity	Treating therapist	16.5	*p* = 0.365	1.70, 0.85	*p* = 0.424
	Observing therapist	20.0		1.55, 0.71	
F Strength training (including electrical stimulation for strength)	Treating therapist	46.0	*p* = 0.271	1.78, 0.74	*p* = 0.087
	Observing therapist	51.5		1.59, 0.80	
G Endurance training (including electrical stimulation for endurance)	Treating therapist	25.0	*p* = 0.012	1.50, 0.71	*p* = 0.841
	Observing therapist	15.0		1.53, 0.73	

SD, standard deviation.

### Influence of therapist, patient, and center characteristics

In addition to assessing agreement between therapists on the intervention, differences between the treating and the observing therapists with respect to discipline, years of experience, location of the center, setting, and patient level/severity of injury were also examined. As shown in Table 3, no differences were identified for these variables regardless of whether the pair consisted of two PTs, two OTs, or a PT/OT.

**Table 3.** Influence of Therapist, Patient, and Center Characteristics

Topic	Groupings	Observations of pairs included	Agreement percentage	Pearson chi-square
Discipline pairings	Both OT	26	76.9	*p* = 0.255
	Both PT	74	87.8	
	OT with PT	100	89.0	
Years of experience	≥3 years	73	90.4	*p* = 0.277
	<3 years	127	85.0	
Degree of impairment	Motor-incomplete tetraplegia	82	86.6	*p* = 0.327
	Motor-complete tetraplegia	26	76.9	
	Motor-incomplete paraplegia	53	88.7	
	Motor-complete paraplegia	39	92.3	
Setting	Inpatient	164	87.8	*p* = 0.470
	Outpatient	36	83.3	
Location	U.S. center	80	83.8	*p* = 0.264
	Non-U.S. center	120	89.2	

OT, occupational therapist; PT, physical therapist.

## Discussion

The intent of this study was to validate the ISCI PT-OT BDS by assessing agreement between pairs of therapists using the ISCI PT-OT BDS to classify category and time of interventions applied during usual clinical practice. In actual use, it is anticipated that the treating PT or OT who is administering therapy would be the one to document the content and time (dose) of therapy. Having an observing therapist also complete the form was a study design strategy to allow comparison of the documentation by two trained therapists based on a single episode of therapy to assess reliability of the form. Therapists were trained to use the paper-based PT-OT BDS form according to the information provided in the syllabus. For future applications, an Android-based app is available.

### Agreement on intervention category and time

The findings of this study support the reliability of the ISCI PT-OT BDS based on percentage agreement on category between pairs of therapists documenting information about the same session. There was high agreement between the treating and the observing therapists for five of the seven intervention categories and medium agreement for the remaining 2 categories (Table 1). For the activity-directed interventions of bed/seated control, standing control, walking/stairs, fine motor UE interventions, and for the impairment-directed strength training interventions, there was agreement of over 75%. However, the agreement between therapist pairs for the activity-directed intervention of gross motor UE training, and impairment-directed category of endurance training did not meet this threshold, with medium agreement of 66% and 50%, respectively.

Documentation of time spent in an intervention had less agreement between the treating and the observing therapist pairs than for category. The likely reason for this is that the observing therapist was instructed to time the intervention with a stopwatch, whereas the treating therapist was not given this instruction. Agreement on time was high for three of the seven intervention categories and medium for four of the seven categories. However, it is valuable to note that for two interventions that achieved only medium agreement the agreement was near 75% (standing, 74.3% and fine motor, 73.2%). Within each category, the close agreement between the percentages for category and time indicates that there were few instances in which there was no agreement on the category and time. Accordingly, the reliability between therapist pairs as indicated by ICC values was moderate to good for all interventions with the exception of gross UE training and endurance training, for which ICC values indicated poor reliability.

In the syllabus and in the training sessions, instructions indicated that if an intervention could logically fall into both an activity-related category and an impairment-directed category, then the therapist should document that intervention in the activity-related category only. As the treating therapist is deciding on the intervention based on an intent that is known to him or her, but not known to the observing therapist, it is possible that the observing therapist defaulted to selecting an activity-directed goal when unsure.

### Treating and observing therapist interactions

The treating therapist and the observing therapist selected the same intervention category for the majority of interventions (Table 2). The exception was the endurance training category, for which the observing therapist selected the category significantly less often than the treating therapist. Conversely, the observing therapist chose the bed/seated control activities, gross UE, and strength categories more often than the treating therapist. Although these differences were not significant, it may suggest that these categories are difficult to differentiate for an observer who is not aware of the treating therapist’s intent. It is possible that observing therapists were more likely to document an intervention as strength training when the treating therapist viewed the intervention as endurance training. Alternatively, it is possible that rather than selecting the endurance training category, the observing therapists defaulted to selection of an activity-related category (bed mobility, gross UE training) based on the instructions to select the activity-directed category if an intervention could reasonably be classified as being either activity-directed or impairment-directed.

### Influence of therapist, patient, and center characteristics

We investigated factors that could result in differences between the treating and the observing therapist in documentation using the ISCI PT-OT BDS. These factors included therapist characteristics (professional discipline and years of experience working with individuals with SCI), degree of impairment (tetraplegia/paraplegia, complete/incomplete), therapy setting (inpatient, outpatient), and whether the center was a U.S.-based versus a non-U.S. center. The finding of no difference in the intervention documentation because of any of the factors analyzed indicates that the BDS is reliable for use among both PTs and OTs with different amounts of experience, with patients with SCI across all injury levels and severities, in both inpatient and outpatient therapy settings, and across international SCI centers.

For the purposes of this study, in which we were interested in evaluating the reliability of items in the BDS, the study procedure involved both a treating therapist and an observing therapist. However, in real-world implementation, although there may be instances where there is value in an observer completing the ISCI PT-OT BDS to document the content of a therapy session, in most instances, the expectation is that the treating therapist will be the one to complete the documentation.

### Lessons learned

A key advantage of the ISCI PT-OT BDS is its simplicity compared with other available taxonomies.^10–12^ In this study, the actual time for each of the components of the therapy was documented by the observing therapist. Differences in time between the observing and the treating therapists indicate that in real-world use, it will be important for the treating therapist to time each component of the therapy session. During the course of the study, questions arose from therapists, which indicated additional details in the syllabus or the inclusion of a FAQ section would improve the guidance for clinicians. For example, the information on the documentation related to power wheelchair use, and additional guidance related to differentiating walking versus standing would be valuable.

### Limitations

It is expected that in actual use, the ISCI PT-OT BDS will be completed by the treating therapist. For this reason, assessing the reliability of the BDS via an observing therapist was the only reasonable approach to answer the question about whether the BDS could be used to reliably capture information about the content of a therapy session. Differences in agreement are therefore attributable to the fact that the treating therapist knew the intent of each component of the interventions within a session while the observing therapist could only surmise based on observation. Conversely, the observing therapist timed the various components within a session with a stopwatch, while the treating therapist did not. When there was agreement between therapists, it was not possible to confirm that they were scoring the same period of the therapy session. We did not create video recordings of the sessions, and so it was not possible to review how specific categories were scored.

## Conclusions

There was generally moderate-to-strong agreement between the treating and observing therapists recording content of therapy sessions using the ISCI-PT-OT-BDS v.1.2. The BDS is reliable for use across settings, countries, and with patients of various impairment levels.

## Rigor and Reproducibility

The study was designed based on methods recommended by the International Spinal Cord Injury Data Set Committee.^7^ All participating therapists engaged in an instructional session to standardize the data collection process and to ask any questions they may have had. The treating and observing therapists, whose data were being compared, were blinded to each other’s entries. The data were entered into an electronic database at each of the 10 participating centers. Members of the work group performed random fidelity checks for quality control on 10% of the records based on a random assignment, in which electronic data entries were compared with information entered on the data collection forms.

## Abbreviations Used

AIS: American Spinal Injury Association Impairment Scale
ICC: intraclass correlation coefficient
ISCI: International Spinal Cord Injury
OT: occupational therapy; occupational therapists
PT: physical therapy; physical therapist
PT-OT BDS: Physical Therapy—Occupational Therapy Basic Data Set
SCI: Spinal Cord Injury
SD: standard deviation
UE: upper extremity

## References

[1] Fouad K, Tetzlaff W. Rehabilitative training and plasticity following spinal cord injury. Exp Neurol 2012;235(1):91–99. 21333646 10.1016/j.expneurol.2011.02.009

[2] Sandrow-Feinberg HR, Houle JD. Exercise after spinal cord injury as an agent for neuroprotection, regeneration, and rehabilitation. Brain Res 2015;1619:12–21. 25866284 10.1016/j.brainres.2015.03.052 PMC4540698

[3] García-Alías G, Barkhuysen S, Buckle M, et al. Chondroitinase ABC treatment opens a window of opportunity for task-specific rehabilitation. Nat Neurosci 2009;12(9):1145–1151. 19668200 10.1038/nn.2377

[4] Torres-Espin A, Forero J, Fenrich KK, et al. Eliciting inflammation enables successful rehabilitative training in chronic spinal cord injury. Brain 2018;141(7):1946–1962. 29860396 10.1093/brain/awy128 PMC6022560

[5] Gomes-Osman J, Cortes M, Guest J, et al. A systematic review of experimental strategies aimed at improving motor function after acute and chronic spinal cord injury. J Neurotrauma 2016;33(5):425–438. 26415105 10.1089/neu.2014.3812 PMC4779320

[6] Anderson KD, Field-Fote EC, Biering-Sørensen F, et al. International spinal cord injury physical therapy-occupational therapy basic data set (Version 1.2). Spinal Cord Ser Cases 2020;6(1):74. 10.1038/s41394-020-00323-z PMC7431417 32807768

[7] Biering-Sørensen F, Alexander MS, Burns S, et al. Recommendations for translation and reliability testing of international spinal cord injury data sets. Spinal Cord 2011;49(3):357–360. 21060313 10.1038/sc.2010.153

[8] Rupp R, Biering-Sørensen F, Burns SP, et al. International standards for neurological classification of spinal cord injury: Revised 2019. Top Spinal Cord Inj Rehabil 2021;27(2):1–22. 10.46292/sci2702-1 PMC8152171 34108832

[9] Portney LG, Watkins MP. (2009). Foundations of Clinical Research: Applications to Practice. Pearson/Prentice Hall.

[10] van Langeveld SA, Post MW, van Asbeck FW, et al. Reliability of a new classification system for mobility and self-care in spinal cord injury rehabilitation: The spinal cord injury-interventions classification system. Arch Phys Med Rehabil 2009;90(7):1229–1236. 19577037 10.1016/j.apmr.2008.12.026

[11] Whiteneck G, Cassaway J, Dijkers M, et al. New approach to study the contents and outcomes of spinal cord injury rehabilitation: The SCIRehab Project. J. Spinal Cord Med 2009;32:251–259. 19810627 10.1080/10790268.2009.11760779 PMC2718827

[12] Zanca JM, Turkstra LS, Chen C, et al. Advancing rehabilitation practice through improved specification of interventions. Arch Phys Med Rehabil 2019;100(1):164–171. 30267670 10.1016/j.apmr.2018.09.110

